# Effectiveness of Exergames on Functional Physical Performance in Older Adults with Knee/Hip Osteoarthritis: A Randomized Controlled Trial

**DOI:** 10.3390/jcm14092968

**Published:** 2025-04-25

**Authors:** Claudio Carvajal-Parodi, Cristhian Mendoza, Cristian Alvarez, Adolfo Soto-Martínez, David Ulloa-Díaz, Carlos Jorquera-Aguilera, Francisco Guede-Rojas

**Affiliations:** 1 Escuela de Kinesiología, Facultad de Odontología y Ciencias de la Rehabilitación, Universidad San Sebastián, Lientur #1457, Concepción 4030000, Chile; claudio.carvajal@uss.cl; 2 Programa de Doctorado en Ciencias de la Actividad Física y del Deporte, Campus Puerto Real, Universidad de Cádiz, Avda. República Saharaui s/n, Puerto Real, 11519 Cádiz, Spain; 3 Escuela de Medicina, Facultad de Medicina, Universidad San Sebastián, Concepción 4030000, Chile; cristhian.mendoza@uss.cl; 4 Exercise and Rehabilitation Sciences Institute, School of Physical Therapy, Faculty of Rehabilitation Sciences, Universidad Andres Bello, Santiago 7591538, Chile; cristian.alvarez@unab.cl; 5 Departamento de Kinesiología, Facultad de Medicina, Universidad de Concepción, Concepción 4030000, Chile; adosoto@udec.cl; 6 Department of Sports Sciences and Physical Conditioning, Universidad Católica de la Santísima Concepción, Concepción 4030000, Chile; dulloa@ucsc.cl; 7 Escuela de Nutrición y Dietética, Facultad de Ciencias, Universidad Mayor, Santiago 8580745, Chile; carlos.jorquera@mayor.cl

**Keywords:** osteoarthritis, aging, exergaming, physical functional performance

## Abstract

**Background/Objectives**: Osteoarthritis (OA) is a leading cause of mobility impairment in older adults, yet few studies have explored exergames (EXGs) as a complementary therapy for knee and/or hip OA (KOA/HOA). This study evaluated the effects of integrating EXGs into conventional therapy (CT) on functional mobility. **Methods**: Sixty participants were randomized into an EXG/CT group or a CT-only group. The interventions lasted 10 weeks (3 sessions/week), and the EXGs were selected from the interactive game Ring Fit Adventure (Nintendo Switch^®^, Kyoto, Japan). **Results**: Functional mobility (Timed Up and Go test) significantly improved in the EXG/CT group but not in the CT group. Additionally, lower-limb strength and aerobic endurance increased in the EXG/CT group. No adverse events were reported, and the adherence was high. **Conclusions**: These findings support EXG-based interventions as a viable complement to CT. Future studies should design OA-specific EXGs and include patient subgroups to expand the impact of interventions using virtual systems.

## 1. Introduction

Osteoarthritis (OA) is a disease that represents a significant and growing burden, being one of the leading causes of chronic pain, mobility loss, and disability in older adults [1]. OA is more prevalent in women, with knee osteoarthritis (KOA) and hip osteoarthritis (HOA) among its most common forms, affecting up to 35.1% and 19.6% of individuals aged ≥50 years, respectively [2]. The substantial impact of OA on physical function underscores the urgent need for innovative treatments to mitigate its consequences.

Standing, walking, and sitting are basic components of functional mobility, which is a key determinant of health and independence in older adults [3]. Reduced mobility increases the risk of disability, hospitalization, and mortality, significantly diminishing quality of life [4]. In the context of OA, joint deterioration affects stability and gait, contributing to locomotor syndrome and increasing dependence on daily activities [5,6]. Additionally, sarcopenia, physical inactivity, and pain further accelerate functional decline [7], highlighting the need for targeted interventions, such as physical exercise programs, to preserve mobility and autonomy in older adults with OA.

Among non-pharmacological treatments for managing KOA and/or HOA (KOA/HOA), physical exercise is widely recognized as a cornerstone intervention that requires careful dosage and application methods [8]. Conventional exercise for KOA/HOA, especially when recommended through a multicomponent approach (including aerobic, resistance, flexibility, balance/coordination, and functional exercises), can reduce symptoms and improve physical function [9]. However, despite its well-documented benefits, participation in exercise programs is often hindered by barriers such as limited physical capacity, fear of falling, pain, poor adherence, social isolation, insufficient professional support, and environmental constraints [10,11]. Therefore, alternatives based on virtual systems and gamification have been proposed as a strategy to enhance the accessibility, engagement, and effectiveness of physical exercise [12].

Exergames (EXGs), a form of physical activity that integrates interactive video games, have emerged as a promising solution to overcome the limitations associated with participation in therapeutic exercise [13]. Especially recommended as a complement to conventional therapy [14,15], EXGs create immersive virtual environments that provide real-time feedback and adapt to patients’ individual needs, fostering positive experiences [16]. Various studies indicate that EXGs can improve clinical and functional outcomes in older adults and individuals with chronic diseases [15,17]; however, despite the growing interest, evidence on their efficacy in specific populations, such as older adults with OA, remains limited.

Most research on EXGs and virtual reality systems for rehabilitation has primarily focused on neurology, while other clinical areas remain underexplored. In this regard, Brepohl and Leite (2023) stated that 14% of investigations have been conducted in orthopedics and traumatology, and only 8% in rheumatology, where OA was included [18]. Specifically addressing OA, the recent literature reviews have examined the multidimensional effects of EXGs. For instance, a systematic review documented that most clinical trials have been carried out on KOA and that these interventions may enhance relevant rehabilitation outcomes such as functional disability, postural balance, muscle strength, proprioception, gait, range of motion, pain, quality of life, depression, and kinesiophobia [19]. Similarly, a meta-analysis focused exclusively on KOA indicated that virtual reality exercise significantly reduces pain while improving muscle strength and functional capacity [20]. It is important to acknowledge that both investigations emphasize the scarcity of studies on OA populations and that research on EXGs has predominantly focused on KOA, without including individuals with HOA. Therefore, considering that OA frequently coexists in multiple joints [21], studying the functional physical performance of individuals with KOA/HOA is novel and represents a contribution to the field. Moreover, the inclusion of patients with HOA in EXG-based interventions is justified due to the pathophysiological and functional similarities between knee and hip OA, such as joint stiffness, muscle weakness, postural instability, and reduced mobility, which may similarly benefit from this type of approach [22]. Based on this background, this study aims to evaluate the effects of integrating EXGs into conventional physical therapy on functional mobility in older adults with KOA/HOA. Additionally, it seeks to examine their influence on other functional fitness measures, including muscular strength, aerobic endurance, and flexibility.

## 2. Materials and Methods

### 2.1. Design and Participants

This study employed a parallel randomized controlled trial design with a 1:1 allocation ratio, ensuring the blinding of the study groups to the outcome assessor and the statistical analyst. One group received EXGs as an adjunct to conventional therapy (EXG/CT group), while the other received conventional therapy alone (CT group). The protocol was approved by the Ethics Committee of the Concepción Health Service (Code 22-12-59) and was registered on ClinicalTrials.gov (ID: NCT05839262) on 15 March 2023.

The study population consisted of patients who were referred to the rehabilitation service of a community primary health center, located in the city of Concepción, Chile. After reviewing the study requirements, participants voluntarily signed an informed consent form in accordance with the Declaration of Helsinki. Patients aged 60 to 84 years, with a diagnosis of mild-to-moderate KOA and/or HOA, based on ACR criteria and a Kellgren–Lawrence radiographic grade of 2 or 3 [23,24], and the ability to walk independently for ≥15 m, were included [25]. The exclusion criteria included decompensated health conditions, the inability to properly use EXGs, the use of opioids or other medications that interfere with the protocol, a Mini-Mental State Examination (MMSE-EFAM) score below 13 [26], a diagnosis of secondary OA, or involvement in other rehabilitation programs within the past three months. Participants were randomly assigned to the study groups using an age-stratified (60–69, 70–79, and 80–84 years) and sex-stratified (male and female) sequence, which was generated by an external professional and concealed in consecutively numbered, opaque, and sealed envelopes until the interventions were assigned by a healthcare center professional. Finally, all experimental procedures, including recruitment and follow-up, were conducted between April 2023 and March 2024.

### 2.2. Intervention Protocols

The intervention sessions were supervised by two physical therapists, and all participants completed a familiarization process before starting the program. Sessions were held three times per week for 10 weeks, with adherence defined as attending at least two-thirds of the scheduled sessions [27]. The exercise plan for both groups was structured into sets and repetitions with an individualized approach, with intensity ranging from light (perceived exertion of 3–4) to moderate (5–6) on a perceived exertion scale of 0 to 10, achieving a weekly exercise volume of 150 min [28]. The perceived exertion scale recommended by the American College of Sports Medicine (ACSM) [28,29] is a subjective tool that allows individuals to self-regulate exercise intensity in real time based on their perception of exertion, with 0 representing “no effort” and 10 indicating “maximum effort”, providing a practical method for estimating physical activity intensity in primary care contexts. During the entire study period, participants were regularly monitored and instructed to avoid participation in any additional structured physical or cognitive training programs while maintaining their usual daily activities.

The protocol for the CT group consisted of administering TENS and hot packs for 10 min, a 5 min general joint mobility warm-up, and 50 min of conventional physical exercise. This comprised aerobic exercises (stationary cycling, step-ups, and treadmill walking), muscle strengthening for the limbs and trunk (elastic bands, dumbbells, and bodyweight exercises), postural balance training (unstable surfaces, single-leg stance, tandem walking, and obstacle walking), and flexibility exercises for the limbs and trunk (controlled stretches alternating standing, sitting, and supine/prone positions on a mat). The session concluded with a 5 min cool-down phase incorporating breathing exercises, followed by an additional 10 min of TENS and hot-pack application.

**Figure 1:**
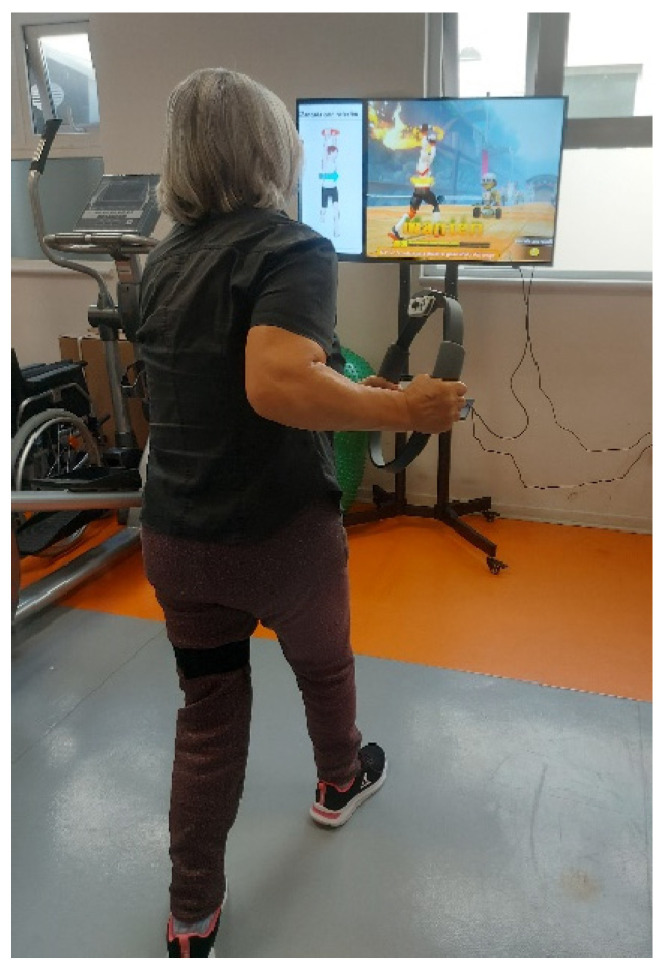
Example of a participant’s interaction with exergames.

In turn, the EXG/CT group protocol followed the same sequence as the CT group, with the difference being that the conventional exercise phase was reduced to 30 min, followed by 20 min of EXGs, maintaining a total exercise time of 50 min per session. To address the same exercise components, 16 EXGs were selected from the video game Ring Fit Adventure, available for the Nintendo Switch^®^ console, and displayed on a 43-inch television (Figure 1). This system was selected due to its accessibility, safety, feasibility, and effectiveness in older adults and patients with chronic pain [30,31], offering appropriate feedback mechanisms. Its commercial availability enhances replicability in community-based or primary care settings. This choice also considered cultural and language compatibility, which facilitated its use and engagement by the participants. These EXGs were alternated from session to session, with six used in each.

The EXGs used consisted of specific exercises, yoga postures, and interactive challenges, which participants performed by following instructions from a virtual guide and adjusting their performance through visual, auditory, and haptic feedback. The EXGs were selected based on specific criteria to ensure their relevance and safety for the target population. The main criteria included the following: (i) targeting at least one component of conventional physical therapy (e.g., muscular strength, aerobic endurance, flexibility, balance, or motor coordination); (ii) allowing for exercise intensity to be adapted based on the user’s perceived exertion; (iii) providing sufficient stability or postural support to minimize fall risk. The set of EXGs selected, their sequence, and the number of repetitions were standardized for all participants. However, the intensity during gameplay was individualized as light to moderate using the same perceived exertion scale (0 to 10) as in the conventional exercises [28,29], allowing real-time adjustments based on each participant’s functional capacity and fatigue level. Table 1 provides a brief description of the EXGs implemented.

### 2.3. Outcome Variables

The primary outcome was functional mobility, assessed using the Timed Up and Go (TUG) test, which measures the time (in seconds) a participant takes to rise from a chair, walk for a distance of 3 m as quickly and safely as possible, turn, and return to a seated position [32]. The shortest time from the three trials, each separated by a one-minute interval, was recorded. The TUG test is a widely used, rapid, and simple clinical tool for assessing mobility and balance, where a longer completion time is associated with prolonged hospitalization, higher mortality, reduced quality of life, less social participation, and difficulties in daily activities [33]; moreover, it has been recently recommended for patients with KOA/HOA [9].

Secondary outcomes were assessed using tests from the Senior Fitness Test (SFT) battery and handgrip strength (HGS). The SFT comprises a set of standardized and reliable tests widely used to provide information on the physical components underlying functional independence in adults aged 60 years and older [34]. The tests that were considered and applied according to the battery manual [35] were as follows: (i) 30 s chair stand: assesses lower-body strength (number of full stands completed in 30 s with arms crossed over the chest); (ii) 30 s arm curl: assesses upper-body strength (number of bicep curls completed in 30 s while holding a hand weight [women 5 lb.; men 8 lb.]); (iii) 2 min step test: assesses aerobic endurance (number of full steps completed in 2 min, raising each knee to a height midway between the patella and iliac crest [score is number of times right knee reaches target]); (iv) chair sit and reach: assesses lower-body flexibility (from sitting position at front of chair, with leg extended and hands reaching toward toes [number of cm (+/−) from extended fingers to tip of toe]); (v) back scratch: assesses upper-body flexibility (with one hand reaching over shoulder and one up middle of back [number of cm (+/−) between extended middle fingers]).

HGS was assessed using a Jamar^®^ Plus + digital dynamometer (Patterson Medical, Warrenville, IL, USA) set to position II, following the protocol established by the American Society of Hand Therapists [36]. The measurement was conducted with the participant seated in a chair without armrests, feet flat on the floor, hips and knees flexed at 90°, shoulder adducted and in neutral rotation, elbow flexed at 90°, forearm in neutral position, and wrist positioned between 15°–30° of extension and 0°–15° of ulnar deviation. Three maximal grip attempts were performed, each lasting 5 s with a 30 s rest interval between trials, and the average of the three measurements was recorded.

Assessments were conducted at baseline (pre-intervention), during the intervention (after sessions 10 and 20), at the end of the intervention (session 30), and four weeks post-intervention as follow-up, with all procedures performed in a designated area of the health center’s therapeutic gymnasium.

### 2.4. Statistical Analysis

Using G*Power 3.1.9.7 software, a sample size of 50 subjects was calculated, assuming a moderate effect size (ES), an alpha level of 0.05, and a statistical power of 0.8; however, 10 additional subjects were included to account for potential dropouts. To provide a more realistic estimation of the intervention effects, data were analyzed under the intention-to-treat approach [37], applying the multiple imputation technique using the predictive mean matching method [38] in SPSS v.25 (IBM Corp., Armonk, NY, USA).

The analysis was conducted in R Studio (2024.09.01) to implement a linear mixed-effects model suitable for longitudinal data with repeated measurements for each subject [39]. The model included fixed effects for group factors (EXG/CT group; CT group) and time (five measurement points), assessing both the main effects and the interactions of the dependent variables. Additionally, a random effect for the subject was added as an intercept to capture individual variability and account for the dependency among repeated measurements. To validate the model, the Shapiro–Wilk test for normality and the Bartlett test for homoscedasticity were performed. Based on the results of these tests, a general linear mixed-effects model (lmer package) was applied when assumptions were met [40], whereas a robust linear mixed-effects model (robustlmm package) was employed when assumptions were violated, enhancing the accuracy of the estimates in the presence of heterogeneity or outliers [39].

Intragroup comparisons were conducted to evaluate changes over time within each group, while intergroup comparisons assessed differences between groups at each measurement point. These analyses were performed using the emmeans package v.1.11.0-004, with the Tukey adjustment for multiple comparisons applied [41]. ESs were calculated using Cohen’s *d* and categorized as negligible (<0.2), small (≥0.2 and ≤0.49), moderate (≥0.5 and ≤0.79), and large (≥0.8), providing additional information on the practical significance of the observed effects [42]. An alpha level of 0.05 was applied for all analyses, and plots were created using GraphPad Prism 9.4.1 (GraphPad Software Inc.; San Diego, CA, USA).

## 3. Results

**Figure 2:**
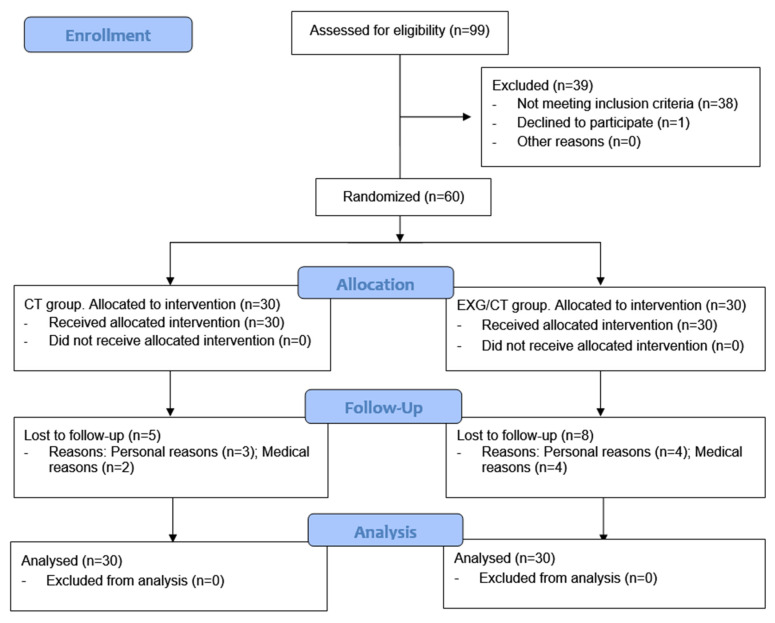
Flowchart of participants during this study.

The CONSORT flowchart in Figure 2 shows that 99 subjects were assessed for eligibility, of whom 60 were recruited and randomized. A total of 13 participants dropped out of this study due to medical and personal reasons unrelated to the intervention. No adverse events were reported, and the adherence was 73.3% in the CT group and 76.7% in the EXG/CT group. Both groups consisted of 25 women and 5 men, and their baseline characteristics are presented in Table 2.

**Figure 3:**
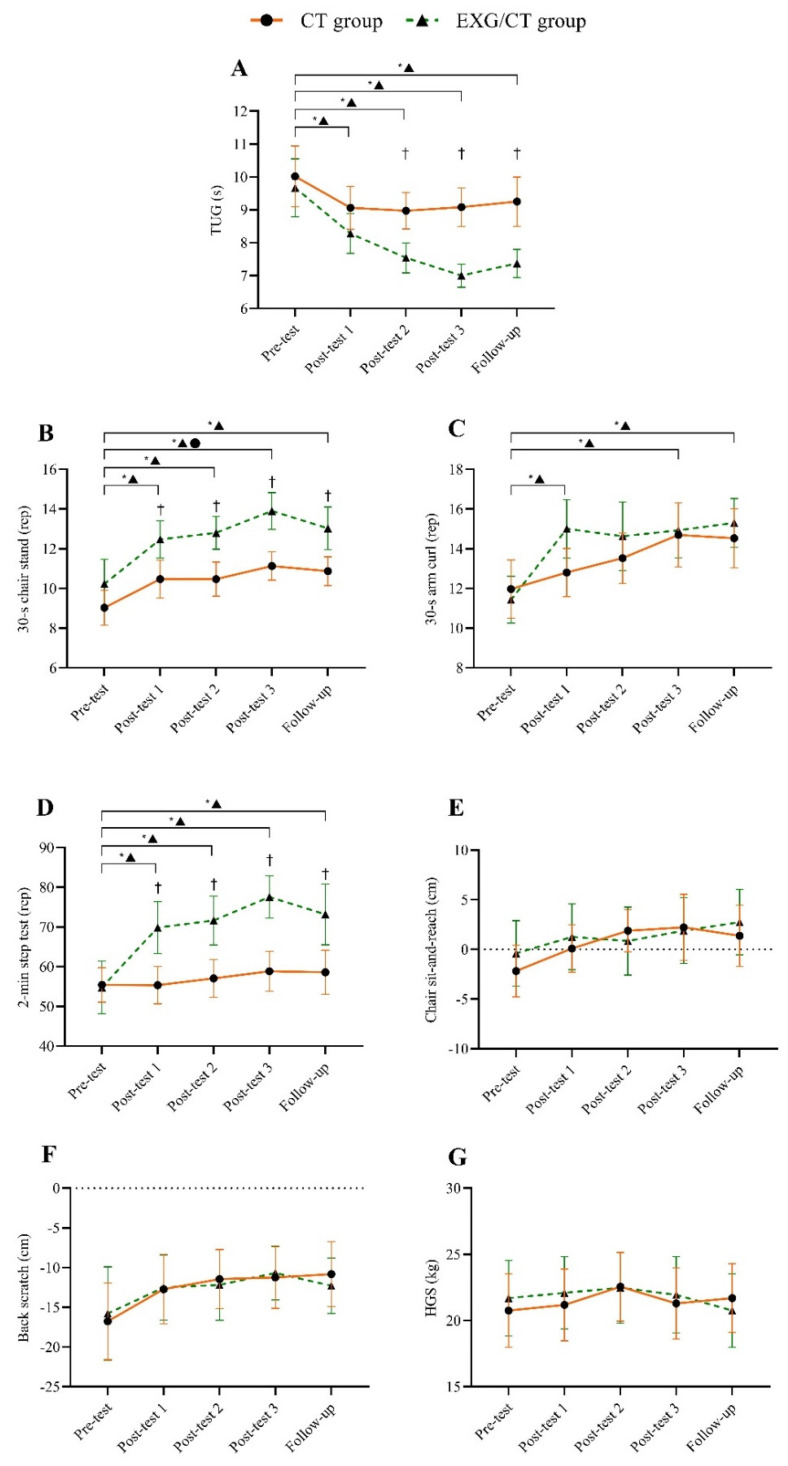
Evolution of the outcome variables throughout this study. (**A**) Timed Up and Go (functional mobility), (**B**) 30 s chair stand (lower-body strength), (**C**) 30 s arm curl (upper-body strength), (**D**) 2 min step test (aerobic endurance), (**E**) chair sit and reach (lower-body flexibility), (**F**) back scratch (upper-body flexibility), and (**G**) hand grip strength. * within-group difference (*p* < 0.05); † between-group difference (*p* < 0.05).

For TUG, the EXG/CT group showed significant improvements in all measurements compared to the pre-test (all *p* < 0.05; d = 0.66 to 1.34). Additionally, there was a significant improvement in post-test 3 compared to post-test 1 (*p* = 0.023; d = 0.93). In contrast, the CT group did not exhibit significant changes throughout this study (all *p* > 0.05). Between-group analyses revealed that the EXG/CT group performed significantly better than the CT group in post-test 2, post-test 3, and follow-up (all *p* < 0.05; d = 1.05 to 1.60). The progression of the TUG test is presented in Figure 3A.

For the 30 s chair stand, 30 s arm curl, and 2 min step test, the EXG/CT group showed significant improvements in all measurements compared to the pre-test (all *p* < 0.05; d = 0.75 to 1.38), except for the 30 s arm curl during post-test 2 (*p* > 0.05). However, no statistically significant changes were observed in the chair sit-and-reach, back scratch, or HGS tests (all *p* > 0.05). The CT group showed a significant improvement only in post-test 3 for the 30 s chair stand compared to the pre-test (*p* < 0.05; d = 0.97). In the between-group analyses, the EXG/CT group demonstrated superior performance in the 30 s chair stand test in post-test 1, post-test 2, post-test 3, and follow-up (all *p* < 0.05; d = 0.78 to 1.25). Similarly, the EXG/CT group outperformed in the 2 min step test across all four time points (all *p* < 0.05; d = 0.81 to 1.34). The progression of these secondary outcome variables is presented in Figure 3B–G.

The statistical measures (means and standard deviations) and effect size categorizations are provided in the Supplementary Material tables.

## 4. Discussion

This study demonstrated that in older adults with mild-to-moderate KOA/HOA, the incorporation of EXGs into conventional physical therapy significantly improved functional mobility compared to conventional therapy alone, with benefits maintained at the one-month follow-up. Greater improvements in muscle strength and aerobic endurance were also observed in the EXG group, while flexibility and HGS showed no changes. The absence of adverse events and the adequate adherence rates denote the safety and feasibility of the interventions. These findings are relevant, as very few clinical trials in this field have been conducted in patients with OA, and most available studies have focused exclusively on KOA [19,20]. Therefore, the novelty of this research lies in providing evidence for both KOA and HOA patients.

The superior and sustained improvements observed in the EXG/CT group compared to the CT group were reflected not only in mean scores but also in predominantly large ESs (Cohen’s *d*) for outcomes such as functional mobility (TUG test), lower-limb muscle strength (30 s chair stand), and aerobic endurance (2 min step test), with these effects being maintained across post-tests and follow-up. This demonstrates the potential of our EXG-based protocol as an adjunct therapeutic modality to conventional therapy. This pattern of improvement aligns with the specificity of the training stimuli applied through EXGs, which emphasized dynamic movements, the activation of large lower-limb muscle groups, and sustained aerobic effort. The observed benefits reflect the physical demands inherent to the EXG activities, supporting their role as a targeted and effective strategy to enhance the core components of functional capacity in older adults with KOA/HOA. It is important to note that no reinforcement or guided practice occurred between the end of the intervention and the follow-up, as participants continued with their usual daily activities, suggesting that the sustained improvements observed may reflect a lasting transfer effect attributable to the intervention.

From a clinical perspective, these effects can be attributed to the ability of EXGs to integrate general and/or specific physical stimuli with multisensory-enriched virtual environments [13]. Moreover, EXGs not only complement the essential components of standard physical exercise but also incorporate a significant cognitive demand, which is crucial in rehabilitation processes [43]. In this regard, Müller et al. (2023) demonstrated that EXGs significantly increase frontal theta activity in older adults compared to reference movements, regardless of game type and difficulty level, underscoring the influence of virtual systems on attentional and executive control processes, which are essential for dual-task performance and cognitive processing in sensorimotor contexts [44].

Contrary to expectations, flexibility and HGS measures did not show significant improvements. From a physiological perspective, this could be explained by the nature of the exercises performed within the clinical context of the studied population, both in conventional and EXG modalities, which may have been less specific and targeted toward these components. Methodologically, it is also possible that the duration and intensity of the intervention were insufficient to produce measurable changes in these specific outcomes. Therefore, considering functional fitness from a comprehensive perspective, it is advisable to review the selected exercises and enhance the inclusion of strategies more directly oriented to flexibility and HGS.

The EXGs used in this study incorporate analytical, global, and functional physical demands, along with visual, auditory, and proprioceptive feedback, while also engaging essential cognitive processes such as sustained attention, decision-making, and motor planning. This multimodal therapeutic approach is crucial for older adults, where functional mobility relies on the integration of coordination, balance, strength, and executive functions [45]. Additionally, the gamification elements present in EXGs appear to enhance motivation and increase engagement in exercise programs, addressing a common challenge in this population [12] while also fostering a range of positive emotions such as happiness, relaxation, self-esteem, self-efficacy, behavioral control, and perceived energy, contributing to a greater enjoyment of therapy [16]. These characteristics establish EXGs as a valuable tool for improving mobility and reinforcing confidence in one’s abilities, promoting independence and quality of life in both older adults [46] and patients with chronic diseases [47]. Thus, integrating EXGs into community clinical settings, as in the present study, could help redefine rehabilitation strategies by providing an accessible and safe option that maximizes the benefits of therapeutic exercise in the target population.

While Ring Fit Adventure is a commercial game originally developed for recreational use, its selection in this study was grounded in prior evidence of feasibility, safety, and acceptability in diverse health-related contexts [30,31]. Nevertheless, its design does not allow for the configuration of joint-specific load control, intensity progression tailored to functional limitations, or personalization for clinical subgroups. These aspects are essential for optimizing rehabilitation outcomes in this population and highlight the opportunity to advance toward the development of EXGs specifically designed for OA, integrating therapeutic precision with the motivational advantages of virtual systems.

In the specific context of KOA/HOA, the results of this study hold particular significance due to the characteristic functional limitations of this population, such as reduced mobility, muscle strength, and postural balance, among others [5,6,8]. The benefits observed in TUG, 30 s chair stand, and the 2 min step test not only reflect an overall improvement in functional capacity but also suggest a positive impact on critical components commonly impaired in these patients. Therefore, the incorporation of EXGs offers an innovative approach by providing a modality that combines physical exercise with cognitive stimuli and sensory feedback to optimize therapeutic outcomes.

The present study considered the TUG test as the primary outcome measure due to its broad recommendation as a tool for functional assessment in the geriatric population [48]. The TUG test is associated with several physical capacity measures. For instance, its performance has been reported to correlate with lower-limb muscle strength, balance, gait speed, and aerobic capacity, all of which are essential components of functional capacity [49]. Moreover, the TUG subtasks (sit-to-stand, walking, and sitting phases) have been shown to correlate with different contractile muscle properties, supporting its use as a more specific tool for functional assessment [50]. The intra- and inter-rater reliability of this test is excellent, with coefficients exceeding 0.95, ensuring precision across various clinical and research applications [51]. Additionally, it has been demonstrated to be sensitive to functional changes following therapeutic interventions, positioning it as a key tool for monitoring disease progression and evaluating treatment effectiveness [3]. Finally, it is a reliable predictor of fall risk in older adults with lower-limb OA, making it particularly relevant for this population [52]. Although the TUG test provides a global measure of functional mobility and does not allow for the identification of specific deficits in components such as balance or muscle strength, its simplicity and widespread international acceptance establish it as a recommended measure for evaluating the effectiveness of interventions in OA patients [9].

In general, our findings on the improvement of functional mobility align with previous studies. A meta-analysis reported a significant reduction in TUG time (MD = −1.46; 95% CI = −2.21 to −0.71; *p* = 0.0001), supporting the role of EXGs in fall prevention and the enhancement of functional capacity in older adults [53]. Similarly, a mixed-methods feasibility study indicated that EXGs can reduce fall risk and improve TUG performance in older adults with KOA, demonstrating their acceptance as a strategy to promote adherence and motivation [54]. Additionally, a clinical trial found that integrating balance training with EXGs amplifies improvements in functional mobility, reinforcing, as in our study, the importance of using mixed intervention approaches [25]. In contrast, Richards et al. (2018) and Sadura-Sieklucka et al. (2023), although not observing changes in TUG performance, identified relevant biomechanical and dynamic balance benefits, emphasizing that virtual biofeedback interventions in KOA may positively influence specific functional components even when their impact on global measures is limited [55,56]. Furthermore, Lee et al. (2023) and Medeiros et al. (2024) pointed out that EXGs are also effective and accessible tools for home-based training, broadening their applicability for populations with limited access to conventional resources [57,58]. These findings, along with our results, support the use of EXGs as a therapeutic option to improve functionality with applications across various settings. However, studies specifically focusing on lower-limb OA remain scarce, reinforcing the need for further research to assess the efficacy of EXGs in this population.

In addition to the benefits observed in functional mobility, our results provide insights into other outcomes, such as muscle strength, aerobic endurance, and flexibility, within a context where the literature reports diverse findings. For example, a meta-analysis reported significant improvements in lower-limb strength but no changes in aerobic endurance, as assessed by the 6 min Walk Test, or in HGS [15]. Another recent review suggests that EXGs may not only be effective in improving gait capacity and balance but also in self-reported functional disability, proprioception, muscle strength, range of motion, pain intensity, and various psychosocial aspects in patients with OA [19]. On the other hand, our results partially align with the clinical trial by Gonçalves et al. (2021) [59], who found that EXGs combined with traditional exercise significantly improved upper- and lower-limb muscle strength; however, functional mobility, assessed with the 8-Foot Up-and-Go test, did not improve. Additionally, compared to the control group, the authors observed better outcomes in upper-limb strength and flexibility [59]. These discrepancies could be attributed to multiple factors, such as differences in intervention volumes and intensities, the virtual systems used, the emphasis on the physical components targeted by EXGs, and the clinical characteristics of the participants, among others. Nevertheless, a fundamental criterion for prescribing EXGs is the functional approach of the exercises and their personalization according to the individual needs of patients [60].

This study has several limitations that should be considered when interpreting the results. First, the intervention was conducted in older adults with mild-to-moderate KOA/HOA, which limits the generalizability of the findings. Although both conditions share basic characteristics such as chronic pain, joint stiffness, reduced mobility, and decreased muscle strength, there may be joint-specific differences in movement patterns, load distribution, and rehabilitation priorities that could influence the response to EXG-based interventions. While this clinical heterogeneity may have affected the precision of the effect estimates, no significant differences were found between groups in the distribution of KOA, HOA, and concomitant KOA and HOA, which minimizes the risk of selection bias and supports the internal validity of our findings. Another limitation is that the EXG system used (Ring Fit Adventure) was not specifically designed for OA rehabilitation, which may have influenced its therapeutic impact. Additionally, the sample size did not allow for detailed subgroup analyses, limiting the identification of variations in treatment responses. The follow-up period lasted four weeks, making it impossible to assess the long-term sustainability of the benefits. Furthermore, no group exclusively receiving EXGs was included, restricting the analysis of the isolated effect of this modality compared to standard treatment. The control of exercise intensity was based on a subjective perceived exertion scale, which, although recommended by the ACSM for older adults, may be influenced by individual variations in effort perception, motivation, or fatigue. This may have affected the accuracy of intensity monitoring during the intervention. Finally, participant preferences for the selected EXGs were not considered, which may have influenced their engagement with the interventions. Beyond these limitations, the strengths of this study lie in its design as a randomized controlled trial with concealed allocation and an intention-to-treat approach, which reinforces the validity of the findings. The inclusion of an active control group allowed for a precise evaluation of the influence of EXGs, while the assessment of multiple functional outcomes provided a comprehensive view of their benefits. Additionally, the field tests used are compatible with the community setting, reinforcing the applicability of the results in primary care rehabilitation settings.

It has been suggested that EXGs represent a promising intervention not only because of their clinical benefits but also due to their economic viability in community-based settings. Compared to conventional interventions, EXGs have shown potential cost effectiveness, particularly when considering improvements in balance, pain reduction, fear of falling, and their high acceptability among older adults [61]. This acceptability, which is key to sustained adherence, translates into a more favorable ratio between implementation costs and achieved health benefits. Moreover, their scalability, suitability for home-based delivery, and potential for remote monitoring strengthen their applicability in health systems with limited resources or in areas with restricted access to in-person services. Integrating EXGs into community programs not only diversifies therapeutic offerings but may also optimize resource use by reducing the need for continuous clinical care. In this sense, their use is not only clinically relevant but also strategically efficient for health systems seeking accessible, sustainable, and patient-centered interventions.

To advance EXG-based interventions, key aspects must be addressed to enhance their applicability in OA rehabilitation. First, studies assessing the isolated effect of EXGs versus standard rehabilitation are needed to establish their specific efficacy, along with long-term follow-ups to determine sustained benefits. Additionally, although this study included patients with both KOA and HOA, future research could focus exclusively on HOA patients to better identify their specific needs, optimize interventions, and develop more targeted recommendations. Moreover, it would be valuable to conduct subgroup analyses considering variables such as age, sex, and comorbidities to identify differences in the effectiveness of EXGs, as well as to design tailored and configurable systems for OA rehabilitation, integrating the functional limitations of this population with biomechanical and clinical principles. In this regard, strategies to adapt EXG interventions for individuals with more severe functional impairments could include simplified movement patterns, seated or assisted formats, and progressive intensity levels. Likewise, patients with lower digital literacy may benefit from more intuitive user interfaces, initial guided sessions, or caregiver assistance to facilitate engagement and adherence.

Furthermore, since moderate-to-large effects were already observed in post-test 1, future research could investigate the feasibility and clinical utility of shorter intervention protocols. This would be especially relevant in community or primary care settings where time and resource constraints may limit the implementation of longer programs. In addition, assessing patient preferences for different types of EXGs could provide critical insights to improve acceptance and adherence, while further research is needed to explore their implementation in different clinical settings, identifying barriers and facilitators to support large-scale adoption.

Finally, it is worth considering that combining EXGs with pharmacological therapies offers a promising multimodal approach to managing OA symptoms. While EXGs may enhance physical and cognitive function, pharmacological treatments may provide additional symptom relief, particularly in more advanced stages of the disease. For example, one study reported early improvements in pain and function following intra-articular injections in patients unresponsive to standard therapies; however, these benefits tend to diminish over time, highlighting the potential need for combined or repeated interventions [62]. In turn, other findings have shown no significant differences between placebo and commonly used injections such as glucocorticoids, hyaluronic acid, or platelet-rich plasma in individuals with mild-to-moderate OA [63]. These data challenge the long-term efficacy of pharmacological monotherapies and reinforce the rationale for integrating EXGs into personalized, multimodal management strategies. Future research should explore the synergistic effects of combining EXGs with pharmacological interventions as part of comprehensive rehabilitation plans.

## 5. Conclusions

This study demonstrates that incorporating EXGs as a complement to conventional physical therapy significantly improves functional mobility, lower-limb muscle strength, and aerobic endurance in older adults with KOA/HOA. Although no significant changes were observed in flexibility and HGS, the observed benefits support the use of EXGs as an innovative therapeutic tool that combines physical and cognitive stimulation in a motivating environment. Future research should focus on the development of EXGs specifically tailored for individuals with OA, incorporating adjustable levels of difficulty, accessible user interfaces, and disease-specific therapeutic goals. Additionally, long-term follow-up studies are needed to assess the sustainability of the observed improvements, explore adherence patterns over time, and determine their potential to prevent physical decline. Evaluating their integration into community-based rehabilitation programs and their effectiveness in diverse clinical subgroups would further enhance their applicability and clinical relevance in real-world settings.

## Figures and Tables

**Table 1:** Exergames selected for this study.

Squats	While keeping feet shoulder-width apart and holding the ring at abdomen level, participants execute slow, controlled squats. An isometric hold is included at the midpoint of the movement to strengthen the lower limbs and improve muscle endurance.
Lateral inclination	Standing with feet aligned at shoulder width and arms raised while holding the ring, the participant performs slow, controlled side bends. An isometric pause at the end range maximizes muscle engagement, promoting flexibility and lateral-trunk stability.
Rotation with inclination	Performed in a semi-squat position with a slight forward lean, this exercise requires alternating trunk rotations while keeping the ring extended in front. At the peak of each turn, the participant holds the position briefly to reinforce core strength and stability.
Lunge with rotation	This exercise combines a forward lunge with a rotational component. As the participant steps into the lunge position, they rotate their torso toward the leading leg while keeping the ring elevated. A brief isometric pause at the peak of the twist enhances core control and balance.
Knee raises	This dynamic movement consists of lifting the knees alternately toward the chest while coordinating arm movements. It promotes lower-limb coordination, activates core muscles, and provides a cardiovascular stimulus.
Squats with extension	In this squat variation, the participant assumes a wider stance with toes slightly turned outward while holding the ring overhead. Squats are performed at a controlled pace, incorporating a brief isometric pause to reinforce lower-limb strength and postural stability.
Dorsal rotation	This exercise involves continuous upper-body twisting from side to side while maintaining an upright posture. The participant stands with feet shoulder-width apart and holds the ring at waist level. The movement enhances spinal flexibility and engages the core muscles.
Crescent moon	In this movement, the participant performs alternating trunk rotations while maintaining a lunge stance and holding the ring in front of the body. The exercise focuses on dynamic stability and core activation.
The chair	This exercise is based on a modified yoga posture where the participant maintains a static half-squat while controlling the movement of the arms through slow flexion and extension. It strengthens the lower body and enhances postural endurance.
The warrior	Inspired by yoga, this movement involves placing the legs in a staggered stance while performing lateral-trunk bends. The ring is held overhead to increase postural engagement and enhance flexibility.
Running path, monster’s lair, jogging bridge	These interactive running games require players to progress through different virtual environments filled with obstacles and challenges. Participants engage in on-the-spot jogging or marching while adjusting their pace, dodging barriers, and responding to in-game prompts. This activity encourages aerobic endurance and dynamic coordination.
Moto adductors	This exercise involves seated gameplay where the participant controls an in-game cart by applying variable pressure on the ring placed between the knees. The goal is to navigate obstacles and collect coins while improving lower-body endurance and control.
Trunk swinging	While holding the ring overhead, the participant performs quick, coordinated trunk movements to collect coins and dodge obstacles. This activity enhances reactivity, agility, and upper-body coordination.
Equilibrism	The objective is to collect coins appearing randomly as the avatar moves along a rail while carrying a horizontal bar. The participant simulates walking in place while holding the ring at waist level and must perform quick lateral-trunk inclinations to avoid obstacles, enhancing coordination and balance.

**Table 2:** Baseline characteristics of study participants.

	CT Group (n = 30)	EXG/CT Group (n = 30)	*p*-Value
Age, years	69.0 ± 5.55	68.73 ± 5.47	0.852
Height, m	1.54 ± 0.07	1.53 ± 0.07	0.621
Weight, kg	72.26 ± 11.09	70.70 ± 12.50	0.612
BMI, kg/m^2^	30.17 ± 4.34	29.82 ± 4.44	0.761
OA diagnosis, no. (%)			
Knee	15 (50)	19 (63.3)	0.298
Hip	6 (20)	7 (23.3)	0.756
Knee and hip	9 (30)	4 (13.3)	0.116

Data are mean ± standard deviation or number (percentage); BMI, body mass index; OA, osteoarthritis; CT, conventional therapy; EXGs, exergames.

## Data Availability

All relevant data are presented in the manuscript. The datasets generated and/or analyzed during the current study are available from the corresponding author upon reasonable request.

## Supplementary Materials

The following supporting information can be downloaded at: https://www.mdpi.com/article/10.3390/jcm14092968/s1, Table S1: Mean, standard deviations and within-group effect sizes; Table S2: Between-group effect sizes.

## Abbreviations

The following abbreviations are used in this manuscript:

OA	Osteoarthritis
KOA	Knee osteoarthritis
HOA	Hip osteoarthritis
EXGs	Exergames
CT	Conventional therapy
TUG	Timed Up and Go
SFT	Senior Fitness Test
HGS	Handgrip strength
ES	Effect size
