# Supplementary material for: Effectiveness of Exergames on Functional Physical Performance in Older Adults with Knee/Hip Osteoarthritis: A Randomized Controlled Trial
Source: J Clin Med. 2025 Apr 25;14(9):2968. doi: 10.3390/jcm14092968 (PMC12072985; doi:10.3390/jcm14092968)
Supplement: Supplementary file 1 [file jcm-14-02968-s001.zip › jcm-3557771-supplementary.pdf]

Supplementary material

Table S1. Mean, standard deviations and within-group effect sizes.

	CT group (n=30)					EXG/CT group (n=30)				
	Pre-test	Post-test 1	Post-test 2	Post-test 3	Follow-up	Pre-test	Post-test 1	Post-test 2	Post-test 3	Follow-up
TUG (s)	10.0 ± 2.4	9.0 ± 1.7<br>d = 0.43 (s)	8.9 ± 1.4<br>d = 0.48 (s)	9.0 ± 1.5<br>d = 0.42 (s)	9.2 ± 2.0<br>d = 0.33 (s)	9.6 ± 2.3	8.2 ± 1.6 *<br>d = 0.66 (m)	7.5 ± 1.23 *<br>d = 1.07 (l)	7.0 ± 0.9 *<br>d = 1.34 (l)	7.3 ± 1.1 *<br>d = 1.19 (l)
30-s chair stand (rep)	9.0 ± 2.3	10.4 ± 2.5<br>d = 0.58 (m)	10.4 ± 2.3<br>d = 0.61 (m)	11.1 ± 1.9 *<br>d = 0.97 (l)	10.8 ± 1.9<br>d = 0.84 (l)	10.2 ± 3.3	12.4 ± 2.5 *<br>d = 0.75 (m)	12.8 ± 2.1 *<br>d = 0.88 (l)	13.9 ± 2.4 *<br>d = 1.23 (l)	13.0 ± 2.8 *<br>d = 0.90 (l)
30-s arm curl (rep)	11.9 ± 3.9	12.8 ± 3.2<br>d = 0.23 (s)	13.5 ± 3.4<br>d = 0.42 (s)	14.7 ± 4.3<br>d = 0.66 (m)	14.5 ± 4.0<br>d = 0.65 (m)	11.4 ± 3.1	15.0 ± 3.9 *<br>d = 0.99 (l)	14.6 ± 4.6<br>d = 0.78 (m)	14.9 ± 3.7 *<br>d = 1.00 (l)	15.3 ± 3.2 *<br>d = 1.20 (l)
2-min step test (rep)	55.4 ± 11.6	55.3 ± 12.6<br>d = 0.01 (n)	57.0 ± 12.7<br>d = 0.13 (n)	58.8 ± 13.4<br>d = 0.27 (s)	58.6 ± 14.8<br>d = 0.23 (s)	54.8 ± 17.8	69.8 ± 17.4 *<br>d = 0.85 (l)	71.6 ± 16.4 *<br>d = 0.98 (l)	77.5 ± 14.3 *<br>d = 1.38 (l)	73.1 ± 20.4 *<br>d = 0.95 (l)
Chair sit-and-reach (cm)	-2.1 ± 6.9	0.1 ± 6.3<br>d = 0.34 (s)	1.8 ± 5.6<br>d = 0.64 (m)	2.2 ± 8.9<br>d = 0.54 (m)	1.3 ± 8.2<br>d = 0.46 (s)	-0.4 ± 8.8	1.2 ± 8.8<br>d = 0.19 (n)	0.8 ± 9.1<br>d = 0.14 (n)	1.8 ± 8.8<br>d = 0.26 (s)	2.7 ± 8.8<br>d = 0.36 (s)
Back scratch (cm)	-16.7 ± 12.9	-12.7 ± 11.6<br>d = 0.32 (s)	-11.4 ± 9.9<br>d = 0.42 (s)	-11.2 ± 10.4<br>d = 0.45 (s)	-10.8 ± 10.9<br>d = 0.49 (s)	-15.7 ± 15.7	-12.5 ± 10.9<br>d = 0.23 (s)	-12.1 ± 11.9<br>d = 0.25 (s)	-10.6 ± 9.0<br>d = 0.37 (s)	-12.2 ± 9.3<br>d = 0.24 (s)
HGS (kg)	20.7 ± 7.4	21.1 ± 7.2<br>d = 0.06 (n)	22.5 ± 6.9<br>d = 0.25 (s)	21.2 ± 7.2<br>d = 0.07 (n)	21.7 ± 6.9<br>d = 0.13 (n)	21.6 ± 7.6	22.0 ± 7.3<br>d = 0.05 (n)	22.4 ± 7.1<br>d = 0.11 (n)	21.9 ± 7.7<br>d = 0.03 (n)	20.7 ± 7.4<br>d = 0.13 (n)

Effect size categories (Cohen's *d*): n negligible, s small, m moderate, l large; * within-group difference (p<0.05); TUG Timed up and go; HGS Hand grip strength.

Table S2. Between-group effect sizes.

	CT group versus EXG/CT group				
	Pre-test, *d*	Post-test 1, *d*	Post-test 2, *d*	Post-test 3, *d*	Follow-up, *d*
TUG	0.145 (n)	0.470 (s)	1.051(l) †	1.605 (l) †	1.181(l) †
30-s chair stand	0.414 (s)	0.787 (m) †	1.039 (l) †	1.251 (l) †	0.880 (l) †
30-s arm curl	0.150 (n)	0.609 (m)	0.270 (s)	0.058 (n)	0.209 (s)
2-min step test	0.042 (n)	0.950 (l) †	0.993 (l) †	1.344 (l) †	0.816 (l) †
Chair sit-and-reach	0.222 (s)	0.152 (n)	0.137 (n)	0.037 (n)	0.161 (n)
Back scratch	0.068 (n)	0.017 (n)	0.066 (n)	0.059 (n)	0.143 (n)
HGS	0.125 (n)	0.125 (n)	0.010 (n)	0.088 (n)	0.132 (n)

Effect size categories (Cohen's *d*): n negligible, s small, m moderate, l large; † between-group difference (p<0.05); TUG Timed up and go; HGS Hand grip strength.
